# Expanding C–T base editing toolkit with diversified cytidine deaminases

**DOI:** 10.1038/s41467-019-11562-6

**Published:** 2019-08-09

**Authors:** Tian-Lin Cheng, Shuo Li, Bo Yuan, Xiaolin Wang, Wenhao Zhou, Zilong Qiu

**Affiliations:** 1 Institute of Neuroscience, State Key Laboratory of Neuroscience, CAS Center for Excellence in Brain Science and Intelligence Technology, Chinese Academy of Sciences, Shanghai, 200031 China; 2 Department of interventional Radiology, Zhongshan Hospital, Fudan University, 180 Fenglin Road, Shanghai, 200032 China; 3 Shanghai Institute of Medical Imaging, Shanghai, 200032 China; 4 Department of Neonatology, Children’s Hospital of Fudan University, Shanghai, 201102 China

**Keywords:** CRISPR-Cas9 genome editing, CRISPR-Cas9 genome editing, CRISPR-Cas9 genome editing

## Abstract

Base editing tools for cytosine to thymine (C–T) conversion enable genome manipulation at single base-pair resolution with high efficiency. Available base editors (BEs) for C–T conversion (CBEs) have restricted editing scopes and nonnegligible off-target effects, which limit their applications. Here, by screening diversified lamprey cytidine deaminases, we establish various CBEs with expanded and diversified editing scopes, which could be further refined by various fusing strategies, fusing at either N-terminus or C–terminus of nCas9. Furthermore, off-target analysis reveals that several CBEs display improved fidelity. Our study expands the toolkits for C–T conversion, serves as guidance for appropriate choice and offers a framework for benchmarking future improvement of base editing tools.

## Abstract (web summary)

Cytosine base editors are limited by editing scope and potential off-target effects. Here the authors screen diversified lamprey cytidine deaminases along with different protein fusion architectures and present base editors with improved fidelity.

## Introduction

The clustered regularly interspaced short palindromic repeat (CRISPR) system is used broadly for genome editing, including gene disruptions and corrections^1^. In principle, CRISPR-associated (Cas) nucleases are recruited by a single guide RNA (sgRNA) onto specific genome target(s) for DNA cleavage, and induced double-stranded DNA breaks (DSBs) are repaired mainly through non-homologous end-joining (NHEJ) pathway, which often results in insertions and deletions (indels), leading to gene inactivation^2,3^. To correct genes carrying damaging mutations, conventionally, exogenous donor DNA templates are needed and homology-directed repair (HDR) pathway is involved^4^. As compared with NHEJ pathway, HDR-dependent gene corrections are usually inefficient. Recently, BEs, which enable nucleotide conversion of specific base pair(s) without DSBs induction, have been developed, through fusing deaminases with Cas nickase (nCas) or catalytically inactive Cas (dCas)^5,6^. As neither donor DNA templates nor DSBs are required, efficiencies of base editing are improved and unwanted indels are reduced, which make BEs attractive tools for functional studies and gene therapy^7^.

Up to now, several cytidine deaminases have been applied for CBEs, including rat APOBEC1 (rAPOBEC1)^5^, human activation-induced deaminase (hAID)^8,9^, apolipoprotein-B-RNA-editing-catalytic polypeptide-like 3A (APOBEC3A)^10^ and *Petromyzon marinus* cytidine deaminase 1 (PmCDA1)^11,12^. These deaminases are fused to either the amino terminus (N-terminus) or carboxy terminus (C–terminus) of nCas9/dCas9 and mainly mediate C–T conversion at positions 4–8 (the PAM was counted as positions 21–23 unless otherwise stated). As prototypical tools exhibit restricted editing scopes, moderate specificity and fidelity, alternative strategies have been tried for improvement, including using Cas9 variants with altered PAM sequences^13–16^ or higher specificity^17^ and different Cas nucleases such as SaCas9^13^ or Cpf1^16,18^. Additionally, strategies such as screening of deaminase mutants^19–21^, SunTag system and MS2 system have also been exploited^8,22^. Recently, circularly permuted Cas9 variants were established, and the editing scopes of BEs were improved greatly when fusing with functional deaminases^16,23^. Though remarkable progresses have been made since then, there is still large room for improvement of targeting scopes, editing specificity and fidelity.

To date, all deaminases used in CBEs are from classical AID/APOBEC family and exhibited similar editing windows. Recently, functional CDA-like (CDAL) deaminases were identified in lampreys displaying significant sequence divergence from classical AID/APOBEC family^24^, making them promising candidates for divergent CBEs with distinct target scopes and specificity.

Here we screen 13 divergent CDAs and CDALs identified in three different lampreys and test their C–T conversion efficiencies as fusing to the N-terminus of nCas9 (D10A) to generate N-terminal CBEs (NT-CBEs). Comparable editing efficiencies are observed for most functional CBEs as compared to reported tools. What’s more, functional CBEs described in this study exhibit shifted and expanded editing scopes, including either narrowed or enlarged editing windows at different positions. Additionally, fusing to the C–terminus of nCas9 (CT-CBEs) further refines the editing scopes of their corresponding NT-CBEs in many cases. Finally, systematic comparisons of editing windows, substrate preference and off-target activities across different CBEs are performed using different sgRNAs, which provide informed choices for appropriate CBEs and offer a framework for benchmarking future improvement of CBEs.

## Results

### Expanded editing scopes with divergent cytidine deaminases

**Fig. 1:**
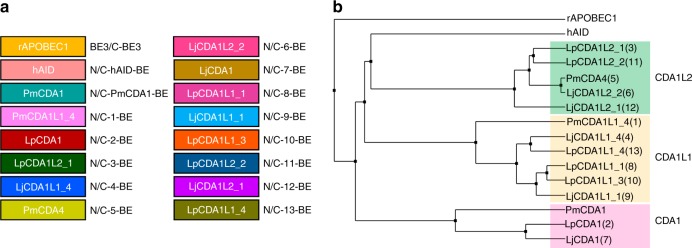
Potential cytidine deaminases for functional CBEs. **a** Cytidine deaminases used in this study for the construction of functional CBEs. Deaminases used for the first time were represented as 1–13. **b** Phylogenetic tree analysis for cytidine deaminases used in this study. It was shown that lamprey cytidine deaminases used here could be classified into three groups: CDA1, CDA1L1, and CDA1L2

**Fig. 2:**
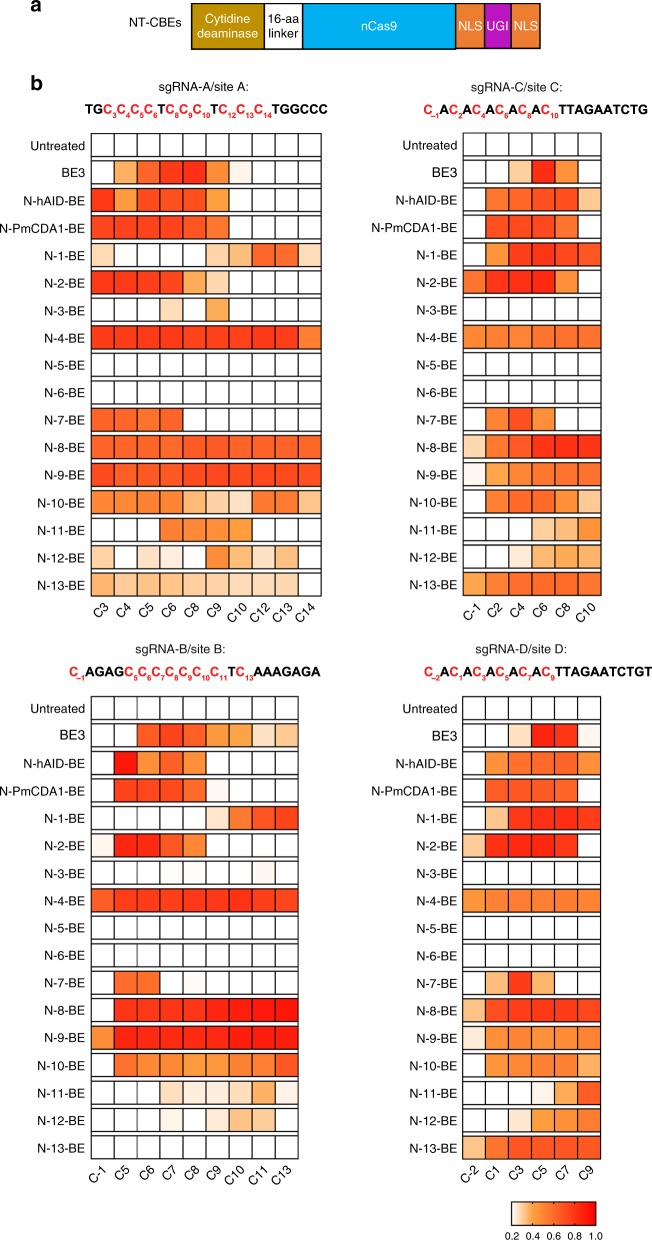
Overview of base editing activities of NT-CBEs. **a** Organization of NT-CBEs. NT-CBEs were generated by fusing different cytidine deaminases to the N-terminus of nCas9. **b** Base editing activities of NT-CBEs against four sgRNAs. sgRNA-A/B/C/D were used and C–T conversion frequencies at every cytosine located in regions 30bp upstream of PAM sites were calculated and heat map showing the editing efficiencies for the critical C sites. Several C sites were not shown because of their low editing efficiencies for all NT-CBEs tested in this study. Data here are represented as mean for three independent experiments. Source data are provided as a Source Data file

Here we selected rAPOBEC1, hAID, PmCDA1 and 13 divergent lamprey CDAs and CDAL deaminases to evaluate their applicability for CBEs^24^ (sequences listed in Supplementary Data 1), and their sequence divergence was analyzed by sequence alignment (Supplementary Fig. 1). Phylogenetic tree analysis showed that lamprey CDAs and CDALs could be classified into three groups (Fig. 1a, b). A panel of N-terminal CBEs (NT-CBEs) was constructed by fusing deaminases to the N-terminus of nCas9 (D10A) (Fig. 2a). To exclude the interference of transfection efficiency and functional editor protein level, as described previously^25^, a two-plasmid system was used for editing activity analysis, with one plasmid expressing CBEs and another plasmid expressing targeting sgRNAs and free UGI to improve base editing efficiency^26^ (Supplementary Fig. 2).

Quantification analysis revealed that 10/13 NT-CBEs containing CDA/CDALs displayed ≥40% C–T conversion at specific C site for ≥2/4 sgRNAs (-A/B/C/D), and their highest editing efficiencies were comparable to NT-CBEs containing rAPOBEC1, hAID and PmCDA1 (Fig. 2b).

### Refined editing scope with C–terminal BEs

**Fig. 3:**
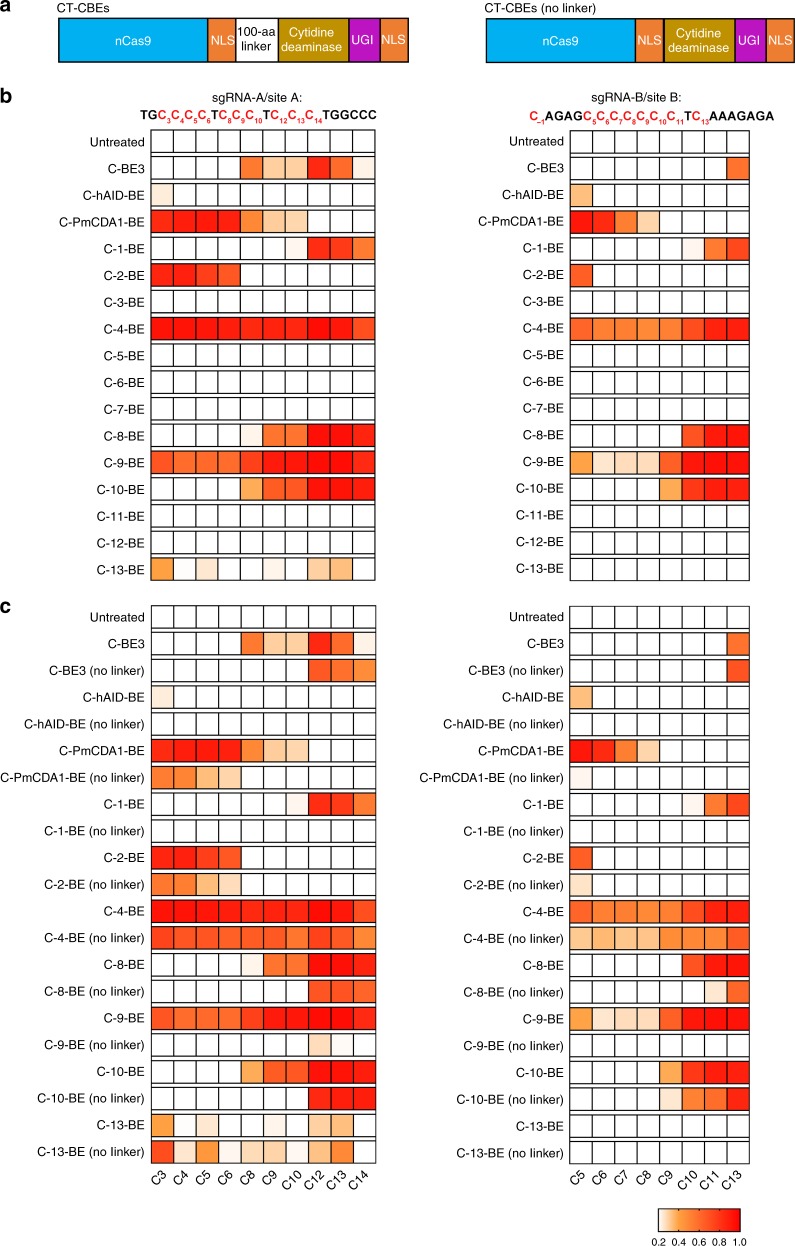
Screening of base editing activities for CT-CBEs with/without the linker. **a** Organization of CT-CBEs. CT-CBEs were generated by fusing different cytidine deaminases to the C–terminus of nCas9. The 100 a.a.–length linker was inserted between nCas9 and cytidine deaminases, which was depleted in CT-CBEs (no linker). **b** Screening for the editing efficiencies of all CT-CBEs with the linker. **c** The linker was depleted in functional CT-CBEs (called CT-CBEs (no linker)) and their editing efficiencies were quantified and compared with the corresponding CT-CBEs. Two sgRNAs, sgRNA-A/B, and Sanger sequencing were used to evaluate the editing activities of CT-CBEs with/without the linker and C–T conversion frequencies at every cytosine located in regions 20bp upstream of PAM sites were quantified with EditR. Heat map showed the editing efficiencies for the critical C sites. Several C sites were not shown because of their low editing efficiencies for all CT-CBEs tested in this study. Source data are provided as a Source Data file

It has been shown that fusing PmCDA1 to the C–terminus of nCas9 generated functional CBEs, and the editing efficiency could be improved with addition of a linker (100 amino acids)^11^. We further constructed sixteen C–terminal BEs (CT-CBEs) with a similar linker (Fig. 3a) and their activities were evaluated using sanger sequencing for two sgRNAs (-A/B). As compared to corresponding NT-CBEs, editing activities of C-7-BE, C-11-BE and C-12-BE were negligible. The activities of C-hAID-BE and C-13-BE were also compromised (Fig. 3b). However, other CT-CBEs, corresponding to functional NT-CBEs, retained >40% editing efficiencies (Fig. 3b). The importance of the linker in functional CT-CBEs was confirmed as linker depletion significantly diminished editing activities for most functional CT-CBEs (Fig. 3a, c). Thereafter, CT-CBEs with the linker (called CT-CBEs unless noted otherwise) were tested in the following experiments.

The applicability of functional CBEs, including NT-CBEs and CT-CBEs, were further evaluated in HEK293T cells and HCT116 cells without sorting process for sgRNA-A/B. Comparable editing efficiencies to BE3 for most functional CBEs were observed, indicating that these divergent CBEs are promising tools for C–T conversion (Supplementary Data 2).

**Fig. 4:**
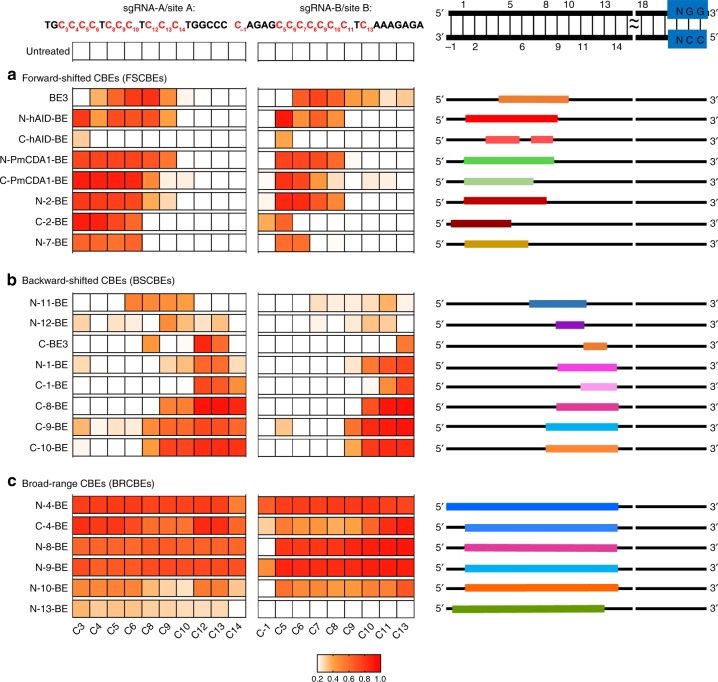
Classifications of CBEs depending on the editing scopes for sgRNA A/B/C/D. The editing scopes were preliminary defined as C positions (CP) with ≥40% editing frequency at sgRNA A/B/C/D. **a** Forward-shifted CBEs (FSCBEs) mainly edit the forward region of target DNA. **b** Backward-shifted CBEs (BSCBEs) mainly edit the backward region of target DNA. **c** Broad-range CBEs (BRCBEs) could edit both forward and backward regions of target DNA. Editing signatures for sgRNA C/D were shown in Supplementary Fig. 3. Source data are provided as a Source Data file

It was noticed that different functional NT- and CT-CBEs displayed diversified editing scopes, which could be classified into three major groups depending on editing signatures of sgRNA-A/B/C/D (C positions (CP) with ≥40% editing frequency). The first group is named forward-shifted CBEs (FSCBEs), which displayed similar editing windows to BE3 (CP4–9), with editing windows at CP1–8/9 for N-hAID-/N-PmCDA1- /N-2-BEs, CP3–5,7–8 for C-hAID-BE, CP1–7 for C-PmCDA1-BE, CP-1–5 for C-2-BE and CP1–6 for N-7-BE (Fig. 4a, Supplementary Fig. 3a). We named the second group backward-shifted CBEs (BSCBEs), displaying editing windows at CP7–11 for N-11-BE, and CP9–11 for N-12-BE, CP11–13 for C-BE3, CP9–14 for N-1-BE, CP11–14 for C-1-BE and CP8/9–14 for C-8/9/10-BE (Fig. 4b, Supplementary Fig. 3b). The third group is named broad-range CBEs (BRCBEs), with editing windows across CP-1–13/14 for N-4/13-BEs and CP1–14 for N-8/9/10-BEs and C-4-BE (Fig. 4c, Supplementary Fig. 3c). We noticed that, as compared to their corresponding NT-CBEs, most functional CT-CBEs displayed narrowed editing scopes while editing scopes of C-BE3 and C-2-BE displayed backward-shifted and forward-shifted manner, respectively.

### Comprehensive editing windows and preference of NT-/CT-CBEs

**Fig. 5:**
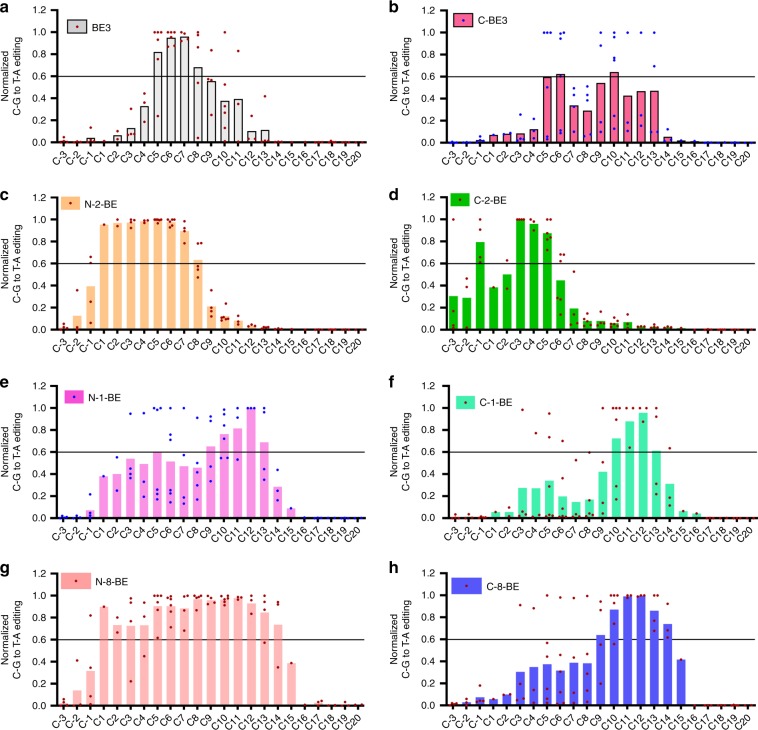
Comprehensive editing windows of functional CBEs. **a**–**h** Normalized C–T conversion efficiency across 9 different sgRNAs for BE3 **a**, C-BE3 **b**, N-2-BE **c**, C-2-BE **d**, N-1-BE **e**, C-1-BE **f**, N-8-BE **g** and C-8-BE **h**. Source data are provided as a Source Data file

To provide a comprehensive and accurate editing scope evaluation, we selected 9 sgRNAs containing multiple and scattered C sites to define the comprehensive editing window (CEW) for representative NT-CBEs and CT-CBEs, including BE3, FSCBEs (N-hAID-BE, N-PmCDA1-BE, C-PmCDA1-BE, N-2-BE, N-7-BE, C-2-BE), BSCBEs (C-BE3, C-hAID-BE, N-1-BE, C-1-BE, N-12-BE, C-8-BE), and BRCBEs (N-4-BE, N-8-BE, N-9-BE, N-10-BE, N-13-BE). We initially compared editing scopes between functional CT-CBEs and corresponding NT-CBEs. In consistent with above results (Fig. 4), the CEW of C-BE3 displayed backward-shifted pattern, and editing efficiency was more average across CP5–13, as compared to BE3 (CP6,10 vs CP5–8) (Fig. 5a, b). C-2-BE, belonging to FSCBEs, displayed forward-shifted pattern as compared to N-2-BE (CP-1&3–5 vs CP1-8) (Fig. 5c, d). For hAID, the CEW of C-hAID-BE was more average as compared to N-hAID-BE (Supplementary Fig. 4a, b). C-PmCDA1-BE displayed narrowed CEW as compared to N-PmCDA1-BE (CP1–6 vs CP1–8) (Supplementary Fig. 4c, d). The CEW of N-7-BE, another member of FSCBE, was CP2–6 (Supplementary Fig. 4e). For BSCBEs, the CEW of C-1-BE was narrowed as compared to N-1-BE (CP10–13 vs CP9–13) (Fig. 5e, f) while N-12-BE CEW was CP7–12 (Supplementary Fig. 4f). For BRCBEs, the CEW of N-8-BE was CP1–14 while the C-8-BE was a member of BSCBEs, with CEW at CP9–14 (Fig. 5g, h). The CEWs of N-4/9/10/13-BEs were quite similar with slight changes (CP-1/1–12/13/15) (Supplementary Fig. 4g–j).

In addition to C positions, sequence context may also affect the editing efficiency. It has been demonstrated that many cytidine deaminases displayed sequence preference. For example, APOBEC1 preferred targeting 5′-AC-3′ and AID preferred 5′-WRC-3′ (W=A/T, R=A/G)^27^. Furthermore, in vitro experiment showed BE1 displayed less preference for 5′-GC-3′^5^. Thus we wondered whether fused cytidine deaminases remained their substrate preference and focused on sequences just upstream or downstream of editable C sites.

We first analyzed cytidine deaminases involved in FSCBEs. It was shown that rAPOBEC1-based CBEs (BE3/C-BE3) displayed minimal efficiency for GC site (underline for target), in consistent with previous in vitro results (Supplementary Fig. 5a). hAID mainly preferred RC (AC/GC>CC/TC) (Supplementary Fig. 5b) while PmCDA1 mainly preferred CA/CC than CG/CT (Supplementary Fig. 5c). LpCDA1 (N/C-2-BE) and LjCDA1 (N-7-BE) showed no obvious preference (Supplementary Fig. 5d, e). For cytidine deaminases of BSCBEs, PmCDA1L1_4 (N/C-1-BE) preferred AC/TC than CC/GC (Supplementary Fig. 5f), while LjCDA1L2_1 (N-12-BE) showed no obvious preference (Supplementary Fig. 5g). For cytidine deaminases of BRCBEs, LpCDA1L1_1 (N/C-8-BE) preferred AC/TC than CC/GC (Supplementary Fig. 5h). LjCDA1L1_4 (N-4-BE) showed less preference for CG (Supplementary Fig. 5i) while LjCDA1L1_1 (N-9-BE) showed no obvious preference (Supplementary Fig. 5j). Additionally, LpCDA1L1_3 (N-10-BE) preferred AC/TC>CC>GC (Supplementary Fig. 5k) while LpCDA1L1_4 (N-13-BE) displayed similar preference to LjCDA1L1_4 (N-4-BE) (Supplementary Fig. 5l). Additionally, the substrate preferences of NT-CBEs and CT-CBEs containing the same deaminase were also evaluated (Supplementary Fig. 6a–l, Supplementary Discussion). More information about substrate preference of NT- and CT-CBEs could be found in Table 1. In addition, it should be noted that editing window shift, genome accessibility and even epigenetic modifications might interfere with the editing efficiency for those similar target sites. As limited sgRNA sites were used in this study, substrate preference described here is still preliminary, and more sgRNAs are needed to clarify this observation.

**Table 1:** Features of different C–T base editors in this study

Name	Deaminase	Editing window	Substrate preference C=edit site	Target Specificity (FAN/HEK4/EMX1)		
BE3	rAPOBEC1	5–8	TC/AC>CC>GC	78%	27%	6%
C-BE3	rAPOBEC1	6,10	TC/AC>CC>GC	83%	28%	3%
N-PmCDA1-BE	PmCDA1	1–8	CA/CC>CG/CT	8%	15%	N.Sp.
C-PmCDA1-BE	PmCDA1	1–6	CA/CC>CG/CT	29%	18%	N.Sp.
N-1-BE	PmCDA1L1_4 (1)	9–13	CA/CT>CG/CC AC/TC>CC/GC	84%	74%	84%
C-1-BE	PmCDA1L1_4 (1)	10–13	CA/CT>CG/CC AC/TC>CC/GC	97%	N.Sp.	—
N-2-BE	LpCDA1 (2)	1–8	N.S.	42%	N.Sp.	N.Sp.
C-2-BE	LpCDA1 (2)	−1,3–5	N.S.	85%	31%	14%
N-4-BE	LjCDA1L1_4 (4)	−1–13	CA/CC/CT>CG;AC>GC	N.Sp.	7%	40%
N-7-BE	LjCDA1 (7)	2–6	CC/CT>CG	81%	61%	68%
N-8-BE	LpCDA1L1_1 (8)	1–14	CA>CG/CC AC/TC>CC/GC	9%	5%	7%
C-8-BE	LpCDA1L1_1 (8)	9–14	CA>CG/CC AC/TC>CC/GC	55%	N.Sp.	58%
N-9-BE	LjCDA1L1_1 (9)	1–15	N.S.	N.Sp.	6%	1%
N-10-BE	LpCDA1L1_3(10)	1–13	CC>CG;AC/TC>CC>GC	85%	24%	47%
N-12-BE	LjCDA1L2_1 (12)	7–12	N.S.	81%	95%	42%
N-13-BE	LpCDA1L1_4 (13)	1–12	CA/CC/CT>CG	24%	31%	43%
N-hAID-BE	hAID	1–8	CA/CC>CT AC/GC>CC/TC	49%	31%	N.Sp.
C-hAID-BE	hAID	3,5,7	CA/CC>CT AC/GC>CC/TC	75%	86%	N.Sp.

N.S.: not significant; N.Sp.: no specificity (higher activity in off-target site than on-target site); — : no obvious activity in on-target site

### Off-target activity analysis for functional NT-/CT-CBEs

**Fig. 6:**
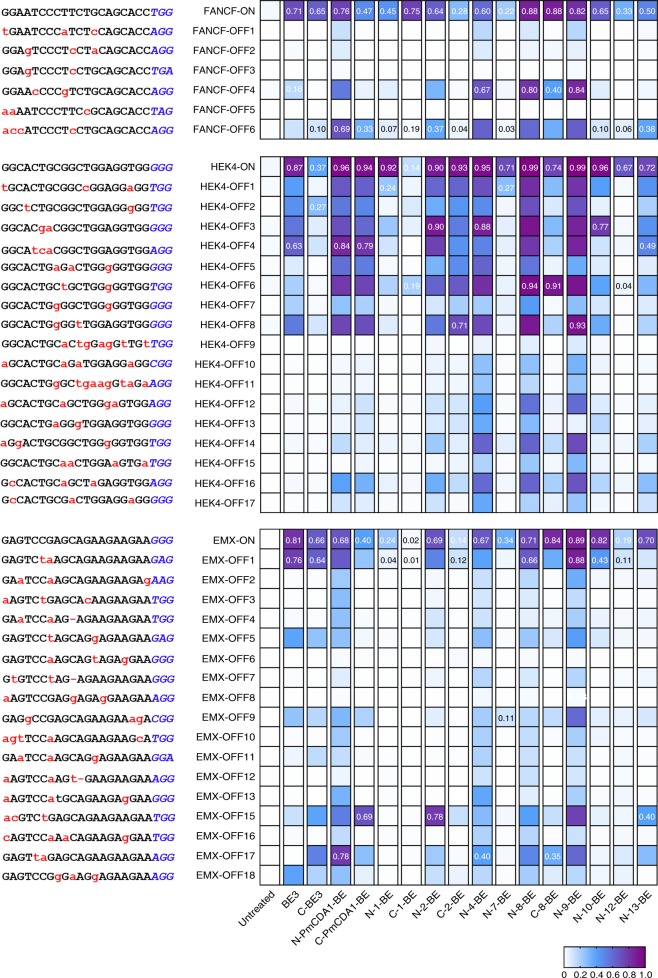
Off-target activities for sgRNA targeting FANCF, HEK4 and EMX across different functional NT-CBEs and CT-CBEs. Heat map of on-target and corresponding off-target efficiencies of representative functional NT-CBEs and CT-CBEs in HEK293T cells was shown. The highest editing efficiencies of different CBEs for each on-target and off-target site were annotated for comparison. Source data are provided as a Source Data file

Off-target activity interfered with functional analysis in biomedical studies and would be damaging for clinical applications, so BEs with minimal off-target activities are desired. It has been shown that CBEs induced detectable C–T conversion events at BE-specific and Cas9-specific off-target loci^5,11,13,28^. To provide comprehensive comparison for the off-target activities across all representative functional NT-CBEs and CT-CBEs, we selected 40 off-target loci, which have been identified previously^28,29^, corresponding to three on-target loci, FANCF, HEK4 and EMX for subsequent analysis. It was shown that rAOPBEC1-based BE3 and C-BE3 displayed high specificity at FANCF site. However, high off-target activities were observed at HEK4 and EMX sites. Moreover, N/C-PmCDA1-BE displayed high off-target activities at all three sites. For other CBEs, we found that N-1-BE, N-7-BE, C-8-BE, and N-12-BE displayed high specificity at ≥2/3 sites (specificity >50%), which indicated that these new CBEs may be better choices under certain conditions (Fig. 6, Supplementary Data 3). In addition, we also noticed that BRCBEs, which possessed the largest editing windows, often displayed high off-target activities.

## Disscussion

Genome manipulation at single base-pair resolution is fascinating, as specific single-nucleotide polymorphisms (SNPs) are common in genomes and associate with many traits and diseases^30^. Indeed, most human pathogenic variations are SNPs, so gene editing at single base-pair resolution is preferred for functional analysis and gene therapies. Though single base editing could be achieved through homologous recombination, complicated operations and low efficiencies limited its applications. Recently, BEs mediating C–T or A–G conversion have emerged^5,6^, providing simplified and efficient tools for single base editing. Efforts have been made to improve editing scope but efficiency and convenience have been compromised. For example, editing windows have been narrowed at the expense of editing efficiency or enlarged at the expense of convenience. Here divergent CBEs using variant cytidine deaminases of lampreys expanded editing scopes of C–T conversion greatly. These functional CBEs were classified into three major types depending on the features of editing windows. The first type called FSCBEs, mainly edited C sites at positions distal to PAM, similar to existed BEs such as BE3 and Target-AID; the second type called BSCBEs, mainly targeted C sites at positions proximal to PAM. Though base editor “BE-PLUS” has been developed to expand editing window width using SunTag system^22^, it is a two-plasmid system needing two functional proteins working together while our tools could achieve more precise C–T conversions with just one functional protein. The third type called BRCBEs, could edit C sites at most positions (CP1-15) of protospacer region with high efficiency. Though most BRCBEs displayed poor specificities, their potent large-scale editing abilities made them suitable for the development of improved base editors with more restricted editing windows through mutant screening or other strategies. Out of BRCBEs, N-9-BE deserved special attention, as it displayed the most significant off-target activities at so many off-target sites and showed no sequence preference. Though N-9-BE may not be suitable for base editing in vivo, it might be re-purposed for 5-methyl- or 5-hydroxymethyl-cytosine detection in vitro, as demonstrated recently^31^. With tools developed in this study, targetable C sites within the genome increased notably and could be further expanded by fusing these functional cytidine deaminases with different CRISPR systems such as SaCas9 and Cpf1^13,18^.

The editing window is critical for the applications of BEs, as it determines which site(s) could be targeted. For CBEs, it is recognized that the position of C site in protospacer region is critical for the editing efficiency of specific BEs. In fact, in addition to the position information, sequence context is also important, as deaminases used for base editing often displayed preference for specific sequences. It has been reported that BE3 exhibited less activity against GC site in vitro^5^, and here we provided in vivo data supported this preference for BE3. We also provided comprehensive substrate preference information for other CBEs, which would guide the choice among these different tools.

In addition to divergent cytidine deaminases from lampreys, this study also emphasized the importance of fusion strategies for C–T conversion. Our results demonstrated that different fusion strategies could impact editing activities, scopes and specificity of many cytidine deaminases. Generally, CT-CBEs are less efficient and several even lost deamination activities as compared to NT-CBEs. Intriguingly, the editing windows of functional CT-CBEs are often narrower and shifted as compared to corresponding NT-CBEs, so CT-CBEs could serve as more precise tools for C–T conversion.

In summary, we provided comprehensive comparison among representative functional CBEs for editing scopes, off-target signatures and substrate preference (Table 1), which would be a valuable guideline for the choice of CBEs. Out of these functional CBEs, N-1-BE, N-7-BE, C-8-BE and N-12-BE displayed comparable editing efficiencies and significantly improved specificity to BE3. Furthermore, N-1-BE, C-8-BE and N-12-BE mainly edit backward regions of target site (CP9–13 vs CP9–14 vs CP7–12), which expanded the targeting scopes of CBEs, making them attractive alternative tools for C–T conversion in future applications.

## Methods

### Cells and cell culture

HEK293T cells (human embryonic kidney epithelial) and HCT116 cells were purchased from Cell Bank of the Chinese Academy of Sciences (Shanghai, China) and cultured in DMEM (Gibco/Life Technologies) with 10% FBS (Gibco/Life Technologies) at 37 °C in a 5% CO_2_ incubator (Thermo Scientific Heraeus).

### Cell transfection

HEK293T cells and HCT116 were plated into 24-well plates (Thermo Scientific) for transfection. About 20 h later, cells were transfected using 2 μL Lipofectamine 3000 (Thermo Scientific) with 2 μg plasmid expressing specific base editor and 500 ng plasmid expressing specific sgRNA according to the manufacturer’s manuals. Cells were cultured for 3 days and culture medium was changed every day. Then cells were isolated and treated with 200 μL lysis buffer (10 mM Tris-HCl, pH 8.8, 5 mM EDTA, 0.2% SDS, 0.2 M NaCl, 25 μg/mL Proteinase K (Calbiochem)) for subsequent genome DNA isolation.

For cell sorting process, HEK293T cells were plated into 12-well plates (Thermo Scientific) for transfection. Cell transfection was performed as described above.

### Phylogeny comparison of cytidine deaminases

Sequence alignment of cytidine deaminases used in this study was generated using MAFFT software with L-INS-I algorithm^32^. Phylogenetic tree was calculated with the percentage identity method implanted in Jalview and constructed using UPGMA tree method.

### Plasmid construction

Plasmid expressing BEs was derived from PX461 (Addgene #48140)^33^. 2A-EGFP cassette in PX461 was replaced with synthesized UGI-2A-EGFP cassette (Genescript). Then EF1alpha promoter was amplified from plasmid MS2-P65-HSF1_GFP (Addgene #61423)^34^, and cloned into NdeI- and NcoI-linearized PX461 to generate EF1alpha-PX461. SgRNA expression cassette was deleted from EF1alpha-PX461 via BsaI and MfeI to generate EF1apha-nCas9 (D10A)-UGI-2A-EGFP plasmid. A linker expressing 16 amino acids was inserted before nCas9 (D10A) for the construction of NT-CBE vectors while another linker expressing the same 100 amino acids of Target-AID was synthesized and inserted after nCas9 (D10A) for the construction of CT-CBE vectors. Additionally, CT-CBE vectors without the linker were also generated.

Human codon optimized deaminases LpCDA1, LpCDA1L1_1/3/4, LpCDA1L2_1/2, LjCDA1, LjCDA1L1_1/4, LjCDA1L2_1/2, PmCDA1L1_4 and PmCDA4 were synthesized commercially (Genescript). Rat APOBEC1, hAID and PmCDA1 were amplified from pCMV-BE3 (Addgene # 73021)^5^, AID-BE3 (Addgene #100803) and CDA1-BE3 (Addgene #100804)^12^, respectively. Detailed sequences for these cytidine deaminases are summarized in Supplementary Data 1.

All deaminases used in this study were amplified and inserted into BamHI- and XbaI- linearized NT-CBE vectors and CT-CBE vectors to generate N-CDA/CDAL-BEs and C-CDA/CDAL-BEs.

For sgRNA expression, nCas9-UGI-2A-EGFP cassette in EF1alpha-PX461 vector was replaced with UGI-2A-mcherry cassette to generate U6-sgRNA-EF1apha-UGI-T2A-mCherry. Oligonucleotides for sgRNAs (sgRNA sequences are listed in Supplementary Table 1) were annealed and inserted into BsaI-linearized pU6-sgRNA-EF1alpha-UGI-2A-mcherry to generate specific sgRNA expression vector.

All subcloning experiments were done using TOP10 competent cells (Tiangen Biotech, Beijing, China)

### Flow cytometry

GFP/mCherry double-positive cells were isolated using a Moflo XDP (Beckman Coulter). In brief, Transfected HEK293T cells were washed with PBS buffer (ThermoFisher Scientific), and digested using 0.05% Trypsin (Gibco) for about 2-3 min. Then culture medium was added to inactivate trypsin and cell suspensions were collected. After centrifugation, cell pellet was resuspended using 200–250 μL PBS for fluorescence-activated cell sorting (FACS) process. Positive cells were sorted into 200 μL lysis buffer (10 mM Tris-HCl, pH 8.8, 5 mM EDTA, 0.2% SDS, 0.2 M NaCl, 25 μg/mL Proteinase K (Calbiochem)) for subsequent genome DNA isolation.

### Sanger sequencing and analysis using EditR

PCR primers about 150bp upstream and downstream of on-target sites were designed and synthesized (Genescript). Then PCR amplification was performed using ~200 ng genomic DNA each with Takara LA Taq. Then sanger sequencing was performed (Shanghai Majorbio Bio-Pharm Technology) and results were quantified using EditR^35^.

### Targeted amplification and high-throughput sequencing

PCR primers about 150bp upstream and downstream of on- and off-targets were designed with different barcodes and synthesized (Genescript). Detailed sequences of these primers are listed (Supplementary Table 1). Then PCR amplification was performed using ~200 ng genomic DNA each with Takara LA Taq. Sequence specificity was verified by agarose gel electrophoresis and then purified using Universal DNA Purification Kit (Tiangen Biotech, Beijing, China). Amplicons with different barcodes were mixed together and DNA library was prepared using NEBNext Ultra II DNA Library Prep Kit (NEB). Deep sequencing was performed using Illumina Hiseq X Ten platform at NovelBio Bio-Pharm Technology, Shanghai, China. All samples were prepared in biological triplicates.

### Base conversion calculation

Raw sequencing reads were demultiplexed initially. Raw data quality was evaluated using FastQC and those with quality score below 15 were trimmed. Data mapping was performed using bowtie 2 (version 2.2.5)^36^ and then piped out with samtools (version 1.3.1)^37^. Base conversion at positions spanning from 30 nt upstream to 10 nt downstream of PAM sites (total 43 bp) was quantified for every base. Base conversion frequencies were calculated as follows: Frequency=base conversion reads/total reads. Detailed data was summarized in Supplementary Data 4.

### Editing window analysis

To examine the editing window regions, cytosine showing the highest C–T conversion frequency in a specified sgRNA was normalized to 1, and other cytosines at positions spanning from 30 nt upstream to 10 nt downstream of PAM sites (total 43 bp) of the same sgRNA were normalized subsequently. Then normalized C–T conversion frequencies were classified and compared according to their positions for all tested sgRNAs of a specified base editor. Comprehensive editing window (CEW) was defined as positions with average C–T conversion efficiency exceeding 0.6 after normalization. SgRNAs used for editing window analysis was listed in Supplementary Table 2.

### Substrate preference analysis

To examine the substrate preference for every cytidine deaminase, C sites were initially classified according to their positions in sgRNA targeting regions and those positions containing at least one C site with ≥0.8 normalized C–T conversion frequency were included in subsequent analysis. Then selected C sites were compared depending on base types upstream or downstream of edited cytosine (NC or CN). For cytidine deaminases showing efficient C–T conversion at both N-terminus and C–terminus of nCas9, the substrate preference was evaluated by integrating respective NT- and CT-CBEs together. For statistical analysis, one-way ANOVA was used and *p* < 0.05 was considered as significant.

### Off target analysis

Editing specificity of CBEs was quantified as follows: on-target C site showing the highest C–T conversion efficiency was defined as onC^h^ while off-target C site showing the highest C–T conversion efficiency was defined as offC^h^. Then the specificity was calculated as (onC^h^ -offC^h^)/ onC^h^ and used to represent editing specificity for the target site.

### Statistical analysis

Statistical details including N, mean and statistical significance values are indicated in the text, figure legends and method details. Error bars in this study represent standard error of the mean (S.E.M) from independent experiments or independent samples. All statistical analysis were performed with GraphPad Prism.

### Reporting summary

Further information on research design is available in the Nature Research Reporting Summary linked to this article.

## Supplementary information

Supplementary Information

Supplementary Data 1

Supplementary Data 2

Supplementary Data 3

Supplementary Data 4

Reporting Summary

Peer Review File

Description of Additional Supplementary Files

Source Data

## Data Availability

The data presented in Figs. 2b, 3b, c, 4a–c, 5a–h and 6, and Supplementary Figs. 3a–c, 4a–j, 5a–l, 6a–l are provided as a source data file. High-throughput sequencing data are available in the National Center for Biotechnology Information Sequence Read Archive database under accession code: PRJNA503988. Processed data are also summarized in Supplementary Data 4. Plasmids and any other relevant data described in this paper are available under reasonable request.

## References

[1] Sander JD, Joung JK (2014). CRISPR-Cas systems for editing, regulating and targeting genomes. Nat. Biotechnol..

[2] Hsu PD, Lander ES, Zhang F (2014). Development and applications of CRISPR-Cas9 for genome engineering. Cell.

[3] Doudna JA, Charpentier E (2014). Genome editing. The new frontier of genome engineering with CRISPR-Cas9. Science.

[4] Cong L (2013). Multiplex genome engineering using CRISPR/Cas systems. Science.

[5] Komor AC, Kim YB, Packer MS, Zuris JA, Liu DR (2016). Programmable editing of a target base in genomic DNA without double-stranded DNA cleavage. Nature.

[6] Gaudelli NM (2017). Programmable base editing of A*T to G*C in genomic DNA without DNA cleavage. Nature.

[7] Rees HA, Liu DR (2018). Base editing: precision chemistry on the genome and transcriptome of living cells. Nat. Rev. Genet..

[8] Hess GT (2016). Directed evolution using dCas9-targeted somatic hypermutation in mammalian cells. Nat. Methods.

[9] Ma Y (2016). Targeted AID-mediated mutagenesis (TAM) enables efficient genomic diversification in mammalian cells. Nat. Methods.

[10] Gehrke JM (2018). An APOBEC3A-Cas9 baseeditor with minimized bystander and off-target activities. Nat. Biotechnol..

[11] Nishida K (2016). Targeted nucleotide editing using hybrid prokaryotic and vertebrate adaptive immune systems. Science.

[12] Komor AC (2017). Improved base excision repair inhibition and bacteriophage Mu Gam protein yields C:G-to-T: Abase editors with higher efficiency and product purity. Sci. Adv..

[13] Kim YB (2017). Increasing the genome-targeting scope and precision of base editing with engineered Cas9-cytidine deaminase fusions. Nat. Biotechnol..

[14] Hu JH (2018). Evolved Cas9 variants with broad PAM compatibility and high DNA specificity. Nature.

[15] Nishimasu H (2018). Engineered CRISPR-Cas9 nuclease with expanded targeting space. Science.

[16] Huang TP (2019). Circularly permuted and PAM-modified Cas9 variants broaden the targeting scope of base editors. Nat. Biotechnol..

[17] Rees HA (2017). Improving the DNA specificity and applicability of base editing through protein engineering and protein delivery. Nat. Commun..

[18] Li X (2018). Base editing with a Cpf1-cytidine deaminase fusion. Nat. Biotechnol..

[19] Grunewald J (2019). Transcriptome-wide off-target RNA editing induced byCRISPR-guided DNA base editors. Nature.

[20] Rees HA, Wilson C, Doman JL, Liu DR (2019). Analysis and minimization of cellular RNA editing by DNA adenine base editors. Sci. Adv..

[21] Zhou C (2019). Off-target RNA mutation induced by DNA base editing and its elimination by mutagenesis. Nature.

[22] Jiang W (2018). BE-PLUS: a new base editing tool with broadened editing window and enhanced fidelity. Cell Res..

[23] Oakes BL (2019). CRISPR-Cas9 circular permutants as programmable scaffolds for genome modification. Cell.

[24] Holland SJ (2018). Expansions, diversification, and interindividual copy number variations of AID/APOBEC family cytidine deaminase genes in lampreys. Proc. Natl Acad. Sci. USA.

[25] Koblan LW (2018). Improving cytidine and adenine base editors by expression optimization and ancestral reconstruction. Nat. Biotechnol..

[26] Wang L (2017). Enhanced base editing by co-expression of free uracil DNA glycosylase inhibitor. Cell Res..

[27] Salter JD, Bennett RP, Smith HC (2016). The APOBEC Protein Family: United by Structure, Divergent in Function. Trends Biochem. Sci..

[28] Kim D (2017). Genome-wide target specificities of CRISPR RNA-guided programmable deaminases. Nat. Biotechnol..

[29] Tsai SQ (2015). GUIDE-seq enables genome-wide profiling of off-target cleavage by CRISPR-Cas nucleases. Nat. Biotechnol..

[30] Landrum MJ (2016). ClinVar: public archive of interpretations of clinically relevant variants. Nucleic Acids Res..

[31] Schutsky EK (2018). Nondestructive, base-resolution sequencing of 5-hydroxymethylcytosine using a DNA deaminase. Nat. Biotechnol..

[32] Katoh K., Rozewicki J., Yamada K. D. MAFFT online service: multiple sequence alignment, interactive sequence choice and visualization. *Brief Bioinform*, 10.1093/bib/bbx108 (2017). 10.1093/bib/bbx108 PMC6781576 28968734

[33] Ran FA (2013). Genome engineering using the CRISPR-Cas9 system. Nat. Protoc..

[34] Konermann S (2015). Genome-scale transcriptional activation by an engineered CRISPR-Cas9 complex. Nature.

[35] KM G (2018). EditR: a method to quantify base editing from sanger sequencing. CRISPR J..

[36] Langmead B, Salzberg SL (2012). Fast gapped-read alignment with Bowtie 2. Nat. Methods.

[37] Li H (2009). The Sequence Alignment/Map format and SAMtools. Bioinformatics.

