# Supplementary material for: Expanding C–T base editing toolkit with diversified cytidine deaminases
Source: Nat Commun. 2019 Aug 9;10:3612. doi: 10.1038/s41467-019-11562-6 (PMC6689024; doi:10.1038/s41467-019-11562-6)
Supplement: Supplementary file 1 — Supplementary Information [file 41467_2019_11562_MOESM1_ESM.pdf]

Expanding C-T base editing toolkit with divergent cytidine deaminases

Cheng et al.

Supplemental Figures

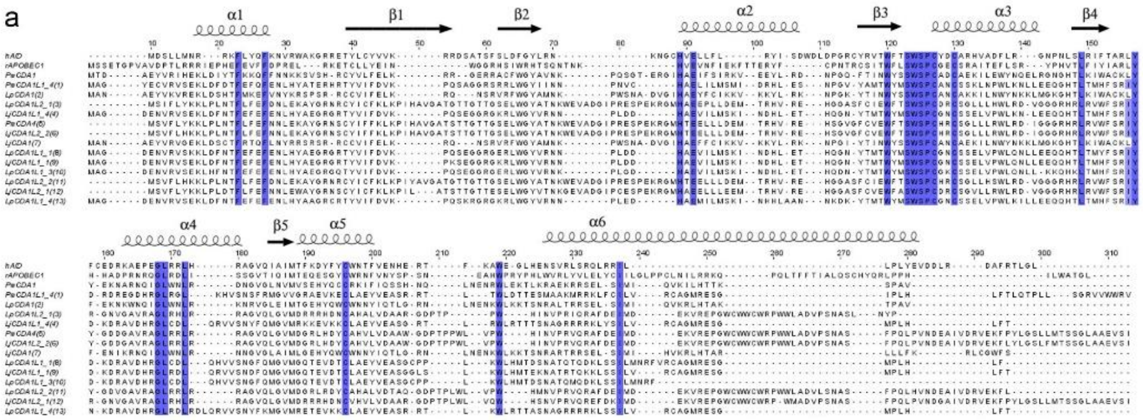

Supplementary Figure 1. Sequence alignment of cytidine deaminases used in this study.

Protein secondary structure of α-helical (α) and β-strand (β) was annotated using hAID structure. Conserved residues for all analyzed cytidine deaminases were colored blue.

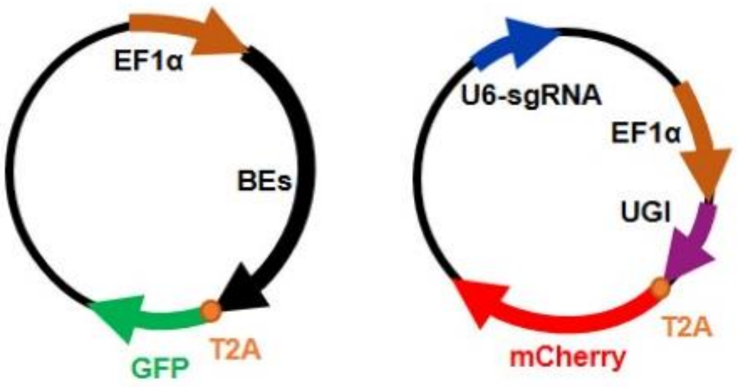

Supplementary Figure 2. Two-plasmid system for editing efficiency analysis. Plasmid expressing base editors could also generate GFP simultaneously via T2A self-cleaving peptide while plasmid expressing sgRNAs contained an UGI-T2A-mcherry cassette generating UGI and mCherry proteins simultaneously.

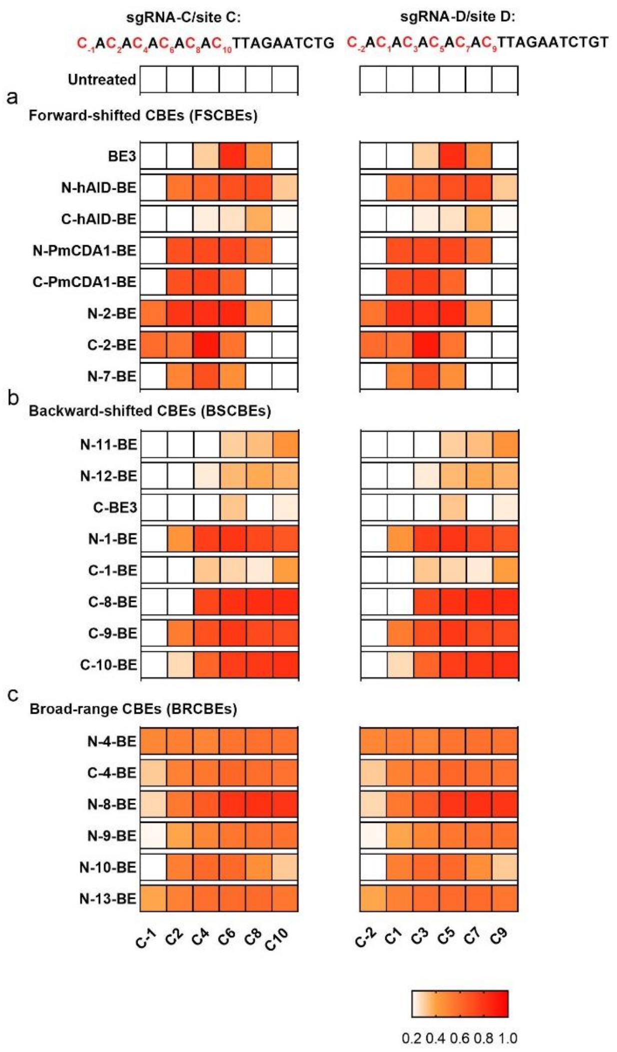

Supplementary Figure 3. The editing signatures of functional CBEs for sgRNA C/D

The editing scopes were preliminary defined as C positions (CP) with ⩾40% editing frequency at sgRNA C/D. (a) Editing signatures of forward-shifted CBEs (FSCBEs) against sgRNA C/D. (b) Editing signatures of backward-shifted CBEs (BSCBEs) against sgRNA C/D. (c) Editing signatures of broad-range CBEs (BRCBEs) against sgRNA C/D. Editing signatures for sgRNA A/B were shown in Figure 4. Source data are provided as a Source Data file.

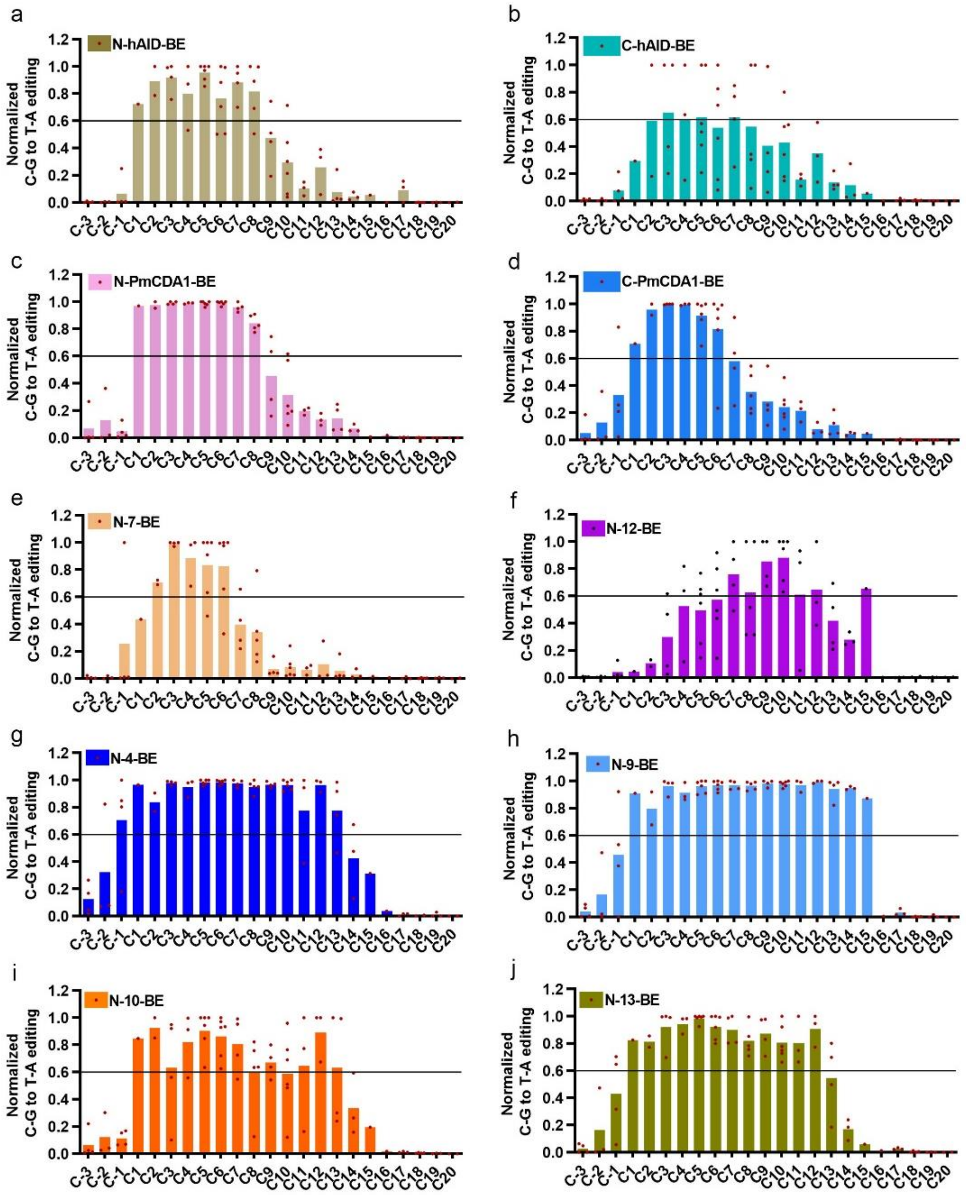

Supplementary Figure 4 Comprehensive editing windows for other functional NT-NBEs and CT-CBEs

Normalized C-T conversion efficiency across 9 different sgRNAs for N-hAID-BE(a), C-hAID-BE(b), N-PmCDA1-BE (c), C-PmCDA1-BE(d), N-7-BE(e), N-12-BE(f), N-4-BE(g), N-9-BE(h), N-10-BE(i) and N-13-BE(j). Source data are provided as a Source Data file.

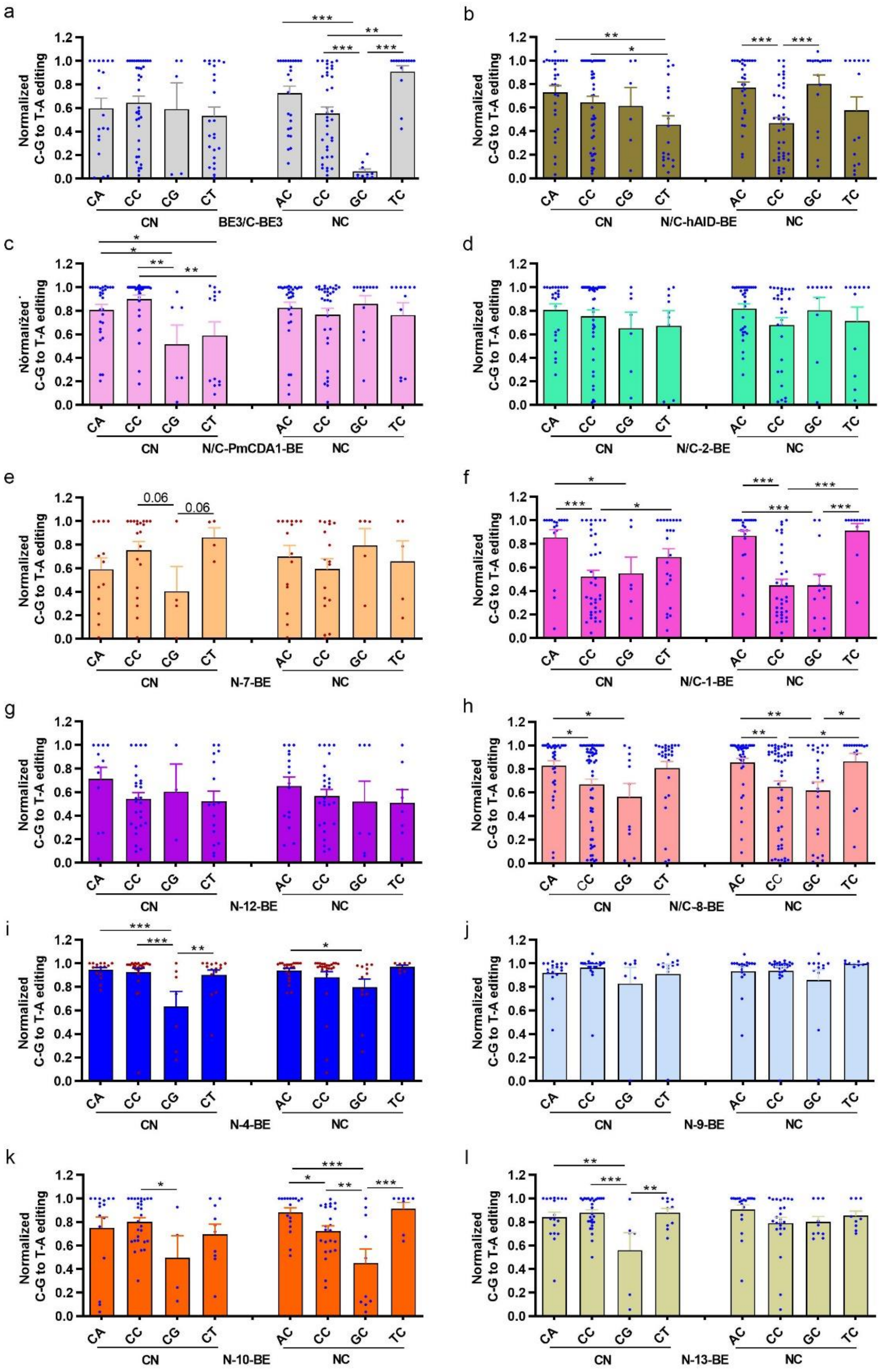

Supplementary Figure 5 Substrate preference for representative NT-CBEs and CT-CBEs

Normalized C-T conversion efficiency across 12 different sgRNAs was classified

according to the downstream and upstream base type for rAPOBEC1 (a), hAID (b), PmCDA1 (c), LpCDA1 (N-2-BE, d), LjCDA1(N-7-BE, e), PmCDA1L1_4(N/C-1-BE, f), LjCDA1L2_1(N-12-BE, g), LpCDA1L1_1(N/C-8-BE, h), LjCDA1L1_4(N-4-BE, i), LjCDA1L1_1(N-9-BE, j), LpCDA1L1_3(N-10-BE, k), LpCDA1L1_4(N-13-BE, l). * $p<0.05$; ** $p<0.01$; *** $p<0.001$. Data here are represented as mean±SEM. Source data are provided as a Source Data file.

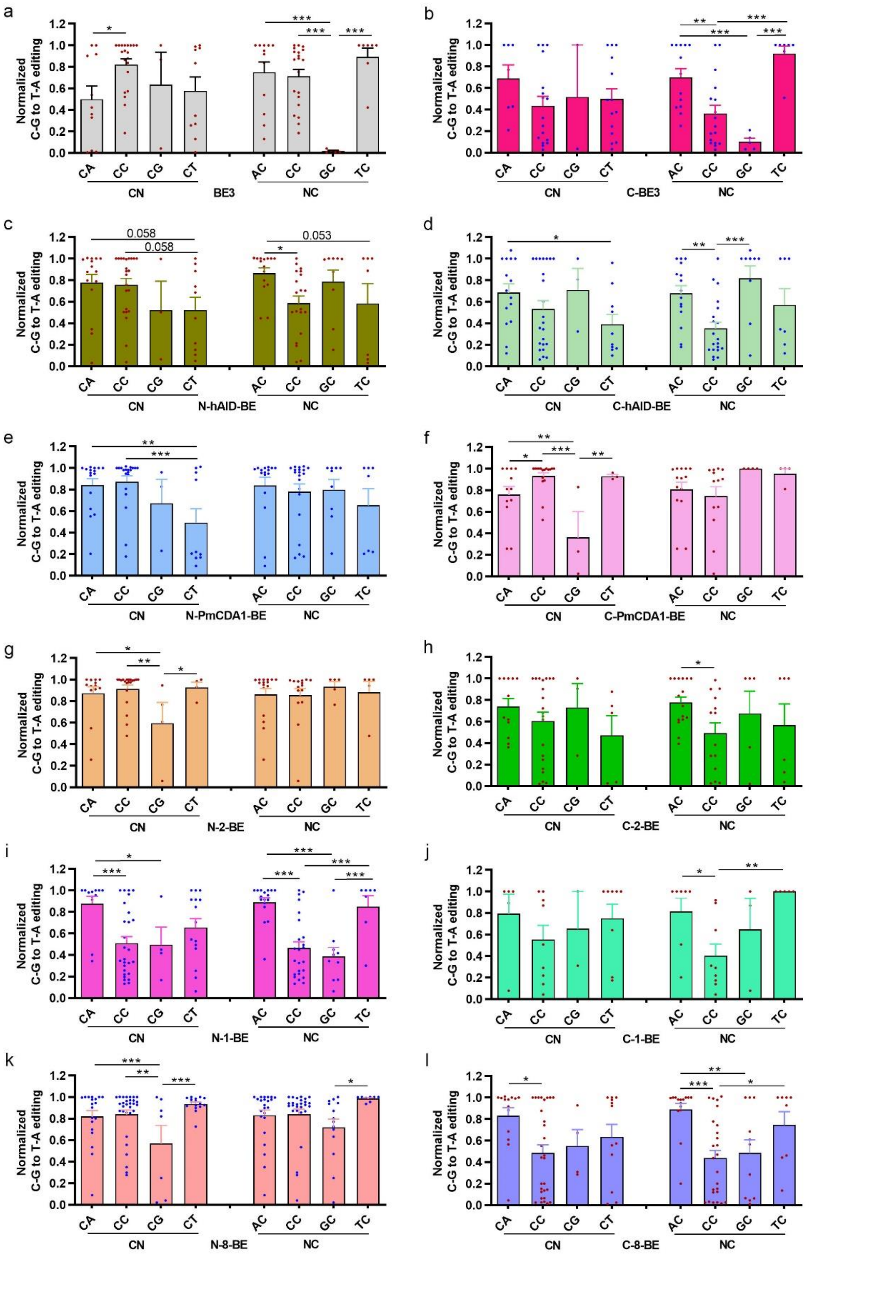

Supplementary Figure 6 Substrate preference for representative NT-CBEs and CT-CBEs containing the same deaminase

Normalized C-T conversion efficiency across 12 different sgRNAs was classified according to the downstream and upstream base type for BE3(a), C-BE3(b), N-hAID-

BE (c), C-hAID-BE (d), N-PmCDA1-BE(e), C-PmCDA1-BE(f), N-2-BE(g), C-2-BE(h), N-1-BE(i), C-1-BE(j), N-8-BE(k), C-8-BE(l). * $p<0.05$; ** $p<0.01$; *** $p<0.001$. Source data are provided as a Source Data file.

Supplementary Table 1: Primers used for PCR amplifications.

sgA/EMX for	CCCATCAGGCTCTCAGCTCAGCC
sgA/EMX rev	CGATGTCCTCCCCATTGGCCTGC
sgB for	GAGGTGCAAAGAGTCCTTGGCAAG
sgB rev	GGCAGTAGTAGGAGGACTTACCCG
sgC for	GAGGTGGAGCTCAAGATCACGTTG
sgC rev	GGTTCTCGCCTGCAGAAAGGTATAG
sgD for	GCATTACCTGGGAGCCTGTTAGA
sgD rev	CAAACTTCAGCGGGCATCAGAA
HEK4 for	GGAACCCAGGTAGCCAGAGAC
HEK4 rev	GCTCCTTTCAACCCGAACGGAG
FAN for	GCCTGGAAGTTCGCTAATCCCGG
FAN rev	GTGAAAGCGGAAGTAGGGCCTTCG
VES2 for	GCGCTGACGGACAGACAGACAG
VES2 rev	GAAGCGAGAACAGCCCAGAAGTTGG
FAN2 for	GTAGGTAGTGCTTGAGACCGCCAG
FAN2 rev	GGAACACGGATAAAGACGCTGGGAG
FAN OFF1 for	GAGACCCTCCTGGTTAAGAGCATG
FAN OFF1 rev	GTGTGTCTGATTGAGTCCCCACAG
FAN OFF2 for	GATGGAGGACAGTGACCCAGG
FAN OFF2 rev	GAGACCTCTGACCTCCACAACTG
FAN OFF3 for	GTCGCAGCTCTCGCACACATAG
FAN OFF4 for	CAGGAGCCGGCTTTCTGTGTC
FAN OFF4 rev	CTCTCAAGTCACCTGGATCGTCC
FAN OFF5 for	GTGATACGGTAATGCTTTCAGCAAAACTG
FAN OFF5 rev	GCATTGAGATGCCAAGTTCCCATAAG
FAN OFF6 for	GCCAGTGAACTAGAGTGGCATG
FAN OFF6 rev	GCCTCACAACTCTGCCATGTG
FAN OFF3 rev	GAGAGCTTCCAGACCCACCTGAAG
HEK4 OFF1 for	CTGAAGATCCCTAGGGGGGCTC
HEK4 OFF1 rev	CTGGCCATTCCGGATGATTCTCC
HEK4 OFF2 for	GTGCAGTGCACTGAAGAGGCTG
HEK4 OFF2 rev	GAAGCCTGTCTTCAGGGCACATG
HEK4 OFF3 for	GTCTGAGGCTCGAATCCTGGCAG
HEK4 OFF3 rev	GAGCAAACCTTGGCATTGTCCCAG
HEK4 OFF4 for	GCTGTGGGATGGAATCACCTG
HEK4 OFF4 rev	GAGTAGAGACAGGCCCAGAGG
HEK4 OFF5 for	CGTGCCTACTGTTGGCGGAGTC
HEK4 OFF5 rev	CTGTACCCTGTGGGTGCTTCAC
HEK4 OFF6 for	CGCTCCGTTGCTTGTCAGCATC
HEK4 OFF6 rev	CACAGAGGAGGCACCACCAGTAC
HEK4 OFF7 for	CTGGCTGTCAGCCCTATCTCC
HEK4 OFF7 rev	CAGATAAGGGTGAGGGGTGGTTAAC

HEK4 OFF8 for	GGTTAAGAGCAGACTCCCTCCTG
HEK4 OFF8 rev	GATTGGAGAGGAGAGCCTGACTG
HEK4 OFF9 for	GTGTCCCATGGAGGCTGCTGG
HEK4 OFF9 rev	GTGGTAGGGACTCACAGGAAGGTG
HEK4 OFF10 for	CAGGAATGAAAGGTTCTGAGGGCGAC
HEK4 OFF10 rev	GTGGGGCACCAGCGTTAGGAAG
HEK4 OFF11 for	GGCCAACTAGAGGCAGACAGGAAG
HEK4 OFF11 rev	GAGTCACTGGAGGCCAGGAACAAG
HEK4 OFF12 for	CCTCAGCACACGACAATTGTGTC
HEK4 OFF12 rev	CTCCAACCTCTTCTAAGCAGCTCC
HEK4 OFF13 for	GGCTCCAGGCAAGTAAAACACCAG
HEK4 OFF13 rev	GCTCCTCATCTTCCGTTGCAGGG
HEK4 OFF14 for	GAAGGTAGCAGAGGAAACTGTGTG
HEK4 OFF14 rev	GCCAGGATAGTCTCGATCTCCTG
HEK4 OFF15 for	GCAGCAGACTCATACAGAACCCAAG
HEK4 OFF15 rev	GGAGTTGACTAGTGCCAGTTCTGG
HEK4 OFF16 for	CATGTATGCAGCTGCTTTTGAATGCC
HEK4 OFF16 rev	GAGGTGGTACTCATTGTTGTTGCAC
HEK4 OFF17 for	GACTTGACGGAGAAAGAGCCCTCG
HEK4 OFF17 rev	GGCAACCCAAAGAGGTTAAGGCTG
EMX OFF1 for	CCATTGAAATCTCACCTGGGCG
EMX OFF1 rev	CAAGGATGCAGTCTCATGACTTGGC
EMX OFF2 for	GAACAATGGCATCAACAGGGAGAGGG
EMX OFF2 rev	GTTGTGCAGTGCACTGTCTGCAGG
EMX OFF3 for	GTTCTGACATTCCTCCTGAGGG
EMX OFF3 rev	CAAACAAGGTGCAGATACAGCAA
EMX OFF5 for	CTGTTTGTCCCTCCACCCTCAAC
EMX OFF5 rev	GTCCTCAATGTGCAGCAGCATCAATG
EMX OFF6 for	GCAGCTGTGCTGTAGAAGACACAG
EMX OFF6 rev	CTTGGCCCCAGTCTCTCTTCTATG
EMX OFF7 for	GGCTTGTCCTCTCAGAGGGTATTGTG
EMX OFF7 rev	CTCACATTACCTGGGTGGCAGACC
EMX OFF8 for	CACCAGGAATACGACTTCCCACATC
EMX OFF8 rev	CAGGGCTGGACTCAAGTCTCC
EMX OFF9 for	GCCATTCATGGAGGGGCACAG
EMX OFF9 rev	GGCTGACCACAAATGCCCAAGAG
EMX OFF10 for	CAAAGCTCTCCCCAGCGTACC
EMX OFF10 rev	CCTCGTCCTGCTCTCACTTAGAC
EMX OFF11 for	CCATAGCAGTGTTATGACAAGGTGCTG
EMX OFF11 rev	GCCTTTCAGGGCCTCAAGTAATCC
EMX OFF12 for	GTGGGGGTGGGAACTAGGCAAG
EMX OFF12 rev	GGTGTGAAGTGGTATCTCACTGTGG
EMX OFF13 for	GCAGCCTGGGCAACAGAGAGAG

EMX OFF13 rev	GGCCAAAATGTTGACCACAGCGTG
EMX OFF14 for	CTGGAGAGCTAGGACCACCTTTCC
EMX OFF14 rev	CAGGCTGATCTCGAACTCCTGACCT
EMX OFF15 for	CTCTCGTCTTCCTGCAGAGGTTC
EMX OFF15 rev	CAGTCAACAAAGCCAGCCTCATAC
EMX OFF16 for	GAAAGCCAAGGGGAATTCCTCTGAT
EMX OFF16 rev	GGAATCTAACTCAGCTGGAAGGCG
EMX OFF17 for	GTGGGGAGATTTGCATCTGTGGAG
EMX OFF17 rev	GGCTTATGGCATGGCAAGACAG
EMX OFF18 for	GAAGGAAGGCAGGAGAGCAAGCAG
EMX OFF18 rev	GAGCAAGGTGGAGGCCCTTGG

Supplementary Table 2: Summarized sgRNA information including sgRNA sequences, locations in DNA strand and positions in PCR products.

sgRNA name	sgRNA sequence	DNA strand	positions in PCR products
sgA	tGCCCCTCCCTCCCTGGCCC	+	77-96
sgB	AGAGCCCCCCCTCAAAGAGA	-	174-191
sgC	ACACACACACTTAGAATCTG	+	141-160
sgD	CACACACACTTAGAATCTGT	+	76-95
HEK4	GGCACTGCGGCTGGAGGTGG	+	164-183
FAN	GGAATCCCTTCTGCAGCACC	-	233-252
EMX	GAGTCCGAGCAGAAGAAGAA	+	165-184
VES2	GACCCCCTCCACCCCGCCTC	-	119-138
FAN2	GCAGAGAGTCGCCGTCTCCA	-	101-120
FAN_OFF1	TGAATCCCATCTCCAGCACCAGG	+	52-71
FAN_OFF2	GGAGTCCCTCCTACAGCACCAGG	+	94-113
FAN_OFF3	GGAGTCCCTCCTGCAGCACCTGA	-	54-73
FAN_OFF4	GGAACCCCGTCTGCAGCACCAGG	-	172-191
FAN_OFF5	aaAATCCCTTCcGCAGCACCTAG	-	65-87
FAN_OFF6	ACCATCCCTCCTGCAGCACCAGG	+	67-86
HEK4_OFF1	TGCACTGCGGCCGGAGGAGGTGG	+	85-104
HEK4_OFF2	GGCTCTGCGGCTGGAGGGGGTGG	-	108-127
HEK4_OFF3	GGCACGACGGCTGGAGGTGGGGG	+	91-110
HEK4_OFF4	GGCATCACGGCTGGAGGTGGAGG	+	60-79
HEK4_OFF5	GGCACTGAGACTGGGGGTGGGGG	-	59-78
HEK4_OFF6	GGCACTGCTGCTGGGGGTGGTGG	-	62-81
HEK4_OFF7	GGCACTGGGGCTGGGGGAGGGGG	+	113-132
HEK4_OFF8	GGCACTGGGGTTGGAGGTGGGGG	-	89-108
HEK4_OFF9	GGCACTGCaCTGGAGGTtGTGG	+	85-106
HEK4_OFF10	aGCACTGCaGaTGGAGGaGGCGG	+	69-91
HEK4_OFF11	GGCACTgGGCTGaAGGTaGAGG	+	115-136
HEK4_OFF12	aGCACTGCaGCTGGgaGTGGAGG	+	128-150
HEK4_OFF13	GGCACTGaGGgTGGAGGTGGGGG	+	163-185
HEK4_OFF14	aGgACTGCGGCTGGgGGTGGTGG	+	157-179
HEK4_OFF15	GGCACTGCaaCTGGAaGTGaTGG	+	153-175
HEK4_OFF16	GcCACTGCaGCTaGAGGTGGAGG	+	114-136
HEK4_OFF17	GcCACTGCGaCTGGAGGaGGGGG	+	105-127
EMX_OFF1	GAGTCtaAGCAGAAGAAGAAGAG	+	116-138
EMX_OFF2	GAaTCCaAGCAGAAGAAGAgAAG	-	73-95
EMX_OFF3	aAGTCtGAGCAcAAGAAGAATGG	-	82-104
EMX_OFF4	GAaTCCaAGAGAAGAAGAATGG	-	36-57
EMX_OFF5	GAGTCCtAGCAGgAGAAGAAGAG	+	218-240
EMX_OFF6	GAGTCCaAGCAGtAGAgGAAGGG	-	61-83

EMX_OFF7	GtGTCCtAGAGAAGAAGAAGGG	+	110-131
EMX_OFF8	aAGTCCGAGgAGAgGAAGAAAGG	-	66-88
EMX_OFF9	GAGgCCGAGCAGAAGAAagACGG	+	150-172
EMX_OFF10	agtTCCaAGCAGAAGAAGcATGG	+	120-142
EMX_OFF11	GAaTCCaAGCAGgAGAAGAAGGA	+	33-55
EMX_OFF12	aAGTCCaAGtGAAGAAGAAAGG	+	130-151
EMX_OFF13	aAGTCCatGCAGAAGAgGAAGGG	+	74-96
EMX_OFF14	GAGTCCtAGAGAAGAAaAAGGG		No PCR product
EMX_OFF15	acGTCtGAGCAGAAGAAGAATGG	-	58-80
EMX_OFF16	cAGTCCaAaCAGAAGAgGAATGG	+	80-102
EMX_OFF17	GAGTTAGAGCAGAAGAAGAAAGG	-	216-238
EMX_OFF18	GAGTCCGGGAAGGAGAAGAAAGG	+	139-161

Supplementary Discussion

Preference differences between NT-CBEs and CT-CBEs containing the same deaminase

For cytidine deaminases showing efficient C-T conversion at both N-terminus and C-terminus of nCas9, we evaluated the substrate preference by comparing NT-CBE with CT-CBEs directly (Supplementary Figure 6), as the substrate preferences are in principle determined by the active-site architectures of the individual deaminase enzymes.

It was shown that the substrate preferences of NT-CBEs and CT-CBEs containing the same deaminase are generally similar with slight differences. BE3 slightly preferred CC than CA (Supplementary Figure 6a) while C-BE3 showed less preference for CC as compared to AC and TC (Supplementary Figure 6b). N-hAID-BE slightly preferred CA/CC than CT whereas C-hAID-BE preferred CA than CT (Supplementary Fig. 6c-d). Furthermore, N-PmCDA1-BE mainly preferred CA/CC than CT (Supplementary Fig. 6e) while C-PmCDA1-BE showed less preference for CG as compared to CA/CC/CT (Supplementary Fig. 6f). N-2-BE, a member of FSBEs, showed less preference for CG as compared to CA/CC/CT (Supplementary Fig. 6g), while corresponding C-2-BE slightly preferred AC than CC (Supplementary Fig. 6h). N-1-BE, a member of BSBEs, preferred AC/TC than CC/GC, and also preferred CA than CC/CG (Supplementary Fig. 6i) while C-1-BE mainly preferred AC/TC than CC (Supplementary Fig. 6g). N-8-BE and corresponding C-8-BE also showed different substrate preference, as N-8-BE preferred CA/CC/CT rather than CG (Supplementary Fig. 6k) while C-8-BE preferred AC/TC than GC/CC, and preferred CA than CC (Supplementary Fig. 6l).

Though different substrate preference was observed for NT-CBEs and CT-CBEs containing the same deaminase, more sgRNAs are needed to confirm this conclusion.

High off-target activities of BRCBEs

It was noticed that BRCBEs displayed broad editing scopes across CP1~14/15, which indicated that they might be more flexible than others to get access to ssDNA. At potential off-target sites, sgRNA-nCas9 complex might induce destabilized and small R-loops, in which ssDNA status are transient and only a small portion of cytidines are accessible. For base editors with low off-target activities, it is difficult to target cytidines in ssDNA of small and destabilized R-loops while BRCBEs are able to edit these transient cytidines in ssDNA.
