# Supplementary material for: Expanding C–T base editing toolkit with diversified cytidine deaminases
Source: Nat Commun. 2019 Aug 9;10:3612. doi: 10.1038/s41467-019-11562-6 (PMC6689024; doi:10.1038/s41467-019-11562-6)
Supplement: Supplementary file 6 — Reporting Summary [file 41467_2019_11562_MOESM6_ESM.pdf]

nature research

Corresponding author(s): Tian-Lin Cheng

Last updated by author(s): Jul 18, 2019

Reporting Summary

Nature Research wishes to improve the reproducibility of the work that we publish. This form provides structure for consistency and transparency in reporting. For further information on Nature Research policies, see Authors & Referees and the Editorial Policy Checklist.

Statistics

For all statistical analyses, confirm that the following items are present in the figure legend, table legend, main text, or Methods section.

n/a	Confirmed	
☐	☒	The exact sample size (n) for each experimental group/condition, given as a discrete number and unit of measurement
☐	☒	A statement on whether measurements were taken from distinct samples or whether the same sample was measured repeatedly
☐	☒	The statistical test(s) used AND whether they are one- or two-sided<br>*Only common tests should be described solely by name; describe more complex techniques in the Methods section.*
☒	☐	A description of all covariates tested
☒	☐	A description of any assumptions or corrections, such as tests of normality and adjustment for multiple comparisons
☐	☒	A full description of the statistical parameters including central tendency (e.g. means) or other basic estimates (e.g. regression coefficient) AND variation (e.g. standard deviation) or associated estimates of uncertainty (e.g. confidence intervals)
☐	☒	For null hypothesis testing, the test statistic (e.g. F, t, r) with confidence intervals, effect sizes, degrees of freedom and P value noted<br>*Give P values as exact values whenever suitable.*
☒	☐	For Bayesian analysis, information on the choice of priors and Markov chain Monte Carlo settings
☒	☐	For hierarchical and complex designs, identification of the appropriate level for tests and full reporting of outcomes
☒	☐	Estimates of effect sizes (e.g. Cohen's d, Pearson's r), indicating how they were calculated

Our web collection on statistics for biologists contains articles on many of the points above.

Software and code

Policy information about availability of computer code

Data collection	FastQC (v0.11.4), bowtie 2 (version 2.2.5), samtools (version 1.3.1)
Data analysis	Raw sequencing reads were demultiplexed initially. Raw data quality was evaluated using FastQC (v0.11.4) and those with quality score below 15 were trimmed. Data mapping was performed using bowtie 2 (version 2.2.5) and then substitution calling was performed with samtools (version 1.3.1).

For manuscripts utilizing custom algorithms or software that are central to the research but not yet described in published literature, software must be made available to editors/reviewers. We strongly encourage code deposition in a community repository (e.g. GitHub). See the Nature Research guidelines for submitting code & software for further information.

Data

Policy information about availability of data

All manuscripts must include a data availability statement. This statement should provide the following information, where applicable:

- Accession codes, unique identifiers, or web links for publicly available datasets
- A list of figures that have associated raw data
- A description of any restrictions on data availability

The data presented in Figs. 2b, 3b-c, 4a-c, 5a-h, and 6 and Supplementary Figs. 3a-c, 4a-j, 5a-l, 6a-l are provided as a source data file. High-throughput sequencing data are available in the National Center for Biotechnology Information Sequence Read Archive database under accession code: PRJNA503988 [https://www.ncbi.nlm.nih.gov/bioproject/PRJNA503988]. Processed data are also summarized in Supplementary Data 4. Plasmids described in this paper are available under reasonable request.

Field-specific reporting

Please select the one below that is the best fit for your research. If you are not sure, read the appropriate sections before making your selection.

☒ Life sciences ☐ Behavioural & social sciences ☐ Ecological, evolutionary & environmental sciences

For a reference copy of the document with all sections, see nature.com/documents/nr-reporting-summary-flat.pdf

Life sciences study design

All studies must disclose on these points even when the disclosure is negative.

Sample size	No statistical methods were used to predetermine sample size. Experiments were performed three times independently unless indicated.
Data exclusions	No data excluded
Replication	Results presented in all figures were reproduced successfully
Randomization	Samples were not randomized
Blinding	The authors were not blinded to group classification

Reporting for specific materials, systems and methods

We require information from authors about some types of materials, experimental systems and methods used in many studies. Here, indicate whether each material, system or method listed is relevant to your study. If you are not sure if a list item applies to your research, read the appropriate section before selecting a response.

Materials & experimental systems

n/a	Involved in the study	
☒	☐	Antibodies
☐	☒	Eukaryotic cell lines
☒	☐	Palaeontology
☒	☐	Animals and other organisms
☒	☐	Human research participants
☒	☐	Clinical data

Methods

n/a	Involved in the study	
☒	☐	ChIP-seq
☐	☒	Flow cytometry
☒	☐	MRI-based neuroimaging

Eukaryotic cell lines

Policy information about cell lines

Cell line source(s)	HEK293T and HCT116 cell lines were from Cell Bank of the Chinese Academy of Sciences
Authentication	No cell lines were authenticated
Mycoplasma contamination	HEK293T cells and HCT116 cells have been confirmed negative for mycoplasma contamination by PCR methods
Commonly misidentified lines (See ICLAC register)	No commonly misidentified cells were used

Flow Cytometry

Plots

Confirm that:

☒ The axis labels state the marker and fluorochrome used (e.g. CD4-FITC).

☒ The axis scales are clearly visible. Include numbers along axes only for bottom left plot of group (a 'group' is an analysis of identical markers).

☒ All plots are contour plots with outliers or pseudocolor plots.

☒ A numerical value for number of cells or percentage (with statistics) is provided.

Methodology

Sample preparation	Cells were cultured and transfected as described in the text and cultured for 3 days. Cells were collected after trypsinization and filtered to remove debris.
Instrument	Moflo XDP (Beckman Coulter)
Software	Summit (version 5.2.0) (Dako Cytomation)
Cell population abundance	Positive cell abundance was dependent on sorting condition and plasmids used. GFP/mCherry double positive cells were typically about 10%-30% of the population.
Gating strategy	Negative control (cells expressing neither GFP nor mCherry fluorescent proteins) and single positive control (cells expressing either GFP or mCherry) were used to establish gates. Gates were drawn to collect cells expressing both GFP and mCherry for subsequent genome extraction, PCR amplification and high-throughput sequencing. Examples were provided for gates used.

☒ Tick this box to confirm that a figure exemplifying the gating strategy is provided in the Supplementary Information.
