# Supplementary material for: Expanding C–T base editing toolkit with diversified cytidine deaminases
Source: Nat Commun. 2019 Aug 9;10:3612. doi: 10.1038/s41467-019-11562-6 (PMC6689024; doi:10.1038/s41467-019-11562-6)
Supplement: Supplementary file 8 — Description of Additional Supplementary Files [file 41467_2019_11562_MOESM8_ESM.docx]

**Title:** Supplementary Data 1
**Description:** Detailed sequences for cytidine deaminases used in this study.

**Title:** Supplementary Data 2
**Description:** Summarized data for base editing in 293T cells and HCT116 cells without cell sorting process.

**Title:** Supplementary Data 3
**Description:** Summarized data for off-target activities in 293T cells for represented CBEs.

**Title:** Supplementary Data 4: Processed data for base editing.”

**Description:** Furthermore, other issues are demonstrated as follows:
