# Supplementary material for: Expanding C–T base editing toolkit with diversified cytidine deaminases
Source: Nat Commun. 2019 Aug 9;10:3612. doi: 10.1038/s41467-019-11562-6 (PMC6689024; doi:10.1038/s41467-019-11562-6)
Supplement: Supplementary file 7 — Peer Review File [file 41467_2019_11562_MOESM7_ESM.pdf]

Editorial Note: This manuscript has been previously reviewed at another journal that is not operating a transparent peer review scheme. This document only contains reviewer comments and rebuttal letters for versions considered at *Nature Communications*.

Reviewers' Comments:

Reviewer #1:
Remarks to the Author:
The authors have adequately addressed all of my concerns regarding their manuscript. The manuscript is qualified for publication in Nature Communications.

Response to reviewers' comments (Comments in italic):

Reviewers' comments:

Reviewer #1:

Remarks to the Author:

The authors have adequately addressed all of my concerns regarding their manuscript. The manuscript is qualified for publication in Nature Communications.

Responses：We thank the reviewer’s contributions to the peer review process of our manuscript and we are glad that the reviewer offered affirmation to our work.
